# Quality indicators for acute cardiovascular diseases: a scoping review

**DOI:** 10.1186/s12913-022-08239-0

**Published:** 2022-07-05

**Authors:** Koshiro Kanaoka, Yoshitaka Iwanaga, Yasushi Tsujimoto, Akihiro Shiroshita, Takaaki Suzuki, Michikazu Nakai, Yoshihiro Miyamoto

**Affiliations:** 1 Department of Medical and Health Information Management, National Cerebral and Cardiovascular Center, Kishibe Shinmaci 6-1, Suita, Osaka, 564-8565 Japan; 2 Department of Nephrology and Dialysis, Kyoritsu Hospital, Kawanishi, Japan; 3 Systematic Review Peer Support Group (SRWS-PSG), Osaka, Japan; 4 Department of Respiratory Medicine, Ichinomiyanishi Hospital, Aichi, Japan; 5 Nara Medical University Library, Nara, Japan; 6 Open Innovation Center, National Cerebral and Cardiovascular Center, Osaka, Japan

**Keywords:** Quality indicator, Scoping review, Acute coronary syndrome, Acute heart failure, Acute aortic dissection

## Abstract

**Background:**

Although many quality indicator (QI) sets have been developed for acute cardiovascular diseases, a comprehensive summary is lacking. In this scoping review we aimed to summarize the available evidence on the QI sets for acute cardiovascular diseases, and assess the QI set development process. We followed the Joanna Briggs Institute framework and the PRISMA extension for scoping reviews.

**Methods:**

We conducted a systematic search of MEDLINE, EMBASE, and major international guidelines on QIs for acute major cardiovascular diseases. The study included articles published after 2000.

**Results:**

Among the 3112 articles screened, 18 were included in this scoping review. Among the 18 articles included, 12 were on acute coronary syndrome (ACS), five on acute heart failure (AHF), and two on acute aortic dissection (AAD); one article included QIs for both ACS and AHF. Only four of these studies conducted a systematic search with a search strategy. From the 18 articles, 268 QIs containing duplication between articles were identified (191 QIs were for ACS, 57 were for AHF, and 20 were for AAD) and QI measurements varied across articles.

**Conclusions:**

This scoping review provides a comprehensive list of QIs for acute cardiovascular diseases. Our results may be helpful to clinicians and organizations seeking to develop QIs for acute cardiovascular care in the future.

**Supplementary Information:**

The online version contains supplementary material available at 10.1186/s12913-022-08239-0.

## Background

Acute cardiovascular diseases, including acute coronary syndrome (ACS), acute heart failure (AHF), and acute aortic dissection (AAD), are common in the general population, and are a leading cause of death worldwide [1, 2]. Nonetheless, well-established care, including the emergency system, invasive treatments, and medical therapies, has reduced mortality from acute cardiovascular diseases. However, the acute-phase mortality rate from acute cardiovascular diseases remains high [3–5]. Moreover, the rate of rehospitalization and long-term mortality varies worldwide [6, 7].

Measurement of the quality of care through quality indicators (QIs) is used to bridge the gap between actual and evidence-based care for patients with cardiovascular diseases. QI sets are commonly developed through the following process: literature review, identification of domains, and selection of the final QI set through a consensus process such as the Delphi method [8]. Some studies have shown that an evidence-practice gap exists in real-world practice, such as early reperfusion in patients with ACS [9, 10]. Furthermore, high attainment of the QI set has been associated with lower risk-adjusted mortality [9, 10]. Although many QI sets are related to acute cardiovascular diseases [11, 12], a comprehensive summary is lacking. Additionally, there may be items that are adopted consistently across different QI sets and others that are unique.

Scoping reviews are a form of knowledge synthesis that incorporates a wide variety of studies to comprehensively summarize and synthesize evidence. This scoping review aimed to summarize the available evidence on the QIs for ACS, AHF, and AAD, and to assess the construction process of these QI sets.

## Methods

### Review of the literature

We conducted a scoping review according to a predefined protocol based on the following five-stage approach developed by the Joanna Briggs Institute (JBI): Stage 1, identifying the research question; Stage 2, identifying relevant studies; Stage 3, study selection; Stage 4, charting the data; and Stage 5, collating, summarizing, and reporting the results [13, 14]. This scoping review followed the preferred reporting items for systematic reviews and meta-analyses extension for scoping reviews statement, and adopted established methodological scoping review frameworks and recommendations [15–17].

### Eligibility criteria

We used the JBI population, concept, and context framework for scoping reviews, to define the inclusion criteria [14]. Our scope was existing literature published after 2000 in which authors built original QIs that focused on acute major cardiovascular diseases (ACS, AHF, and AAD). All published studies that targeted adult (age ≥ 18 years) patients who were hospitalized with acute major cardiovascular diseases diagnosed by physicians were extracted. We reviewed the existing literature that satisfied the following conditions: (1) QIs including any form of quality measures, such as quality metrics and performance measures; (2) QIs assessing any component of Donabedian’s model, namely, any structure (i.e. human resources and hospital equipment), process (i.e., diagnosis and treatment), and outcome measurement (i.e., patient status) [18]; (3) QIs being used in acute care settings; (4) the literature search process (i.e., literature review or systematic review); and (5) a predefined QI creation process (i.e. Delphi or modified Delphi method, or another decision-making process). Studies that assessed or validated the QI sets from existing studies or guidelines were excluded. The context in this review was limited to acute care settings where hospitalization occurred; thus, outpatient and chronic care settings were excluded. There were no restrictions regarding cultural factors, geographic location, race, gender, or particular settings.

### Search strategy and selection of studies

Following the initial limited search, a systematic search was performed across MEDLINE and EMBASE on June 24, 2021 (Supplementary methods). We also checked the reference lists of the included studies, including international guidelines for major cardiovascular diseases. Furthermore, we searched the websites of relevant organizations, including the Agency for Healthcare Research and Quality, the National Institute for Health and Care Excellence, and the Australian Commission on Safety and Quality in Health Care. Only the latest article was included in the analysis if updated QI sets had been reported from the same organization. There were no language restrictions. Conference abstracts, systematic reviews of secondary data of existing studies which were not used for the literature search for QI development, and case studies were excluded, following the predefined protocol [13].

**Fig. 1:**
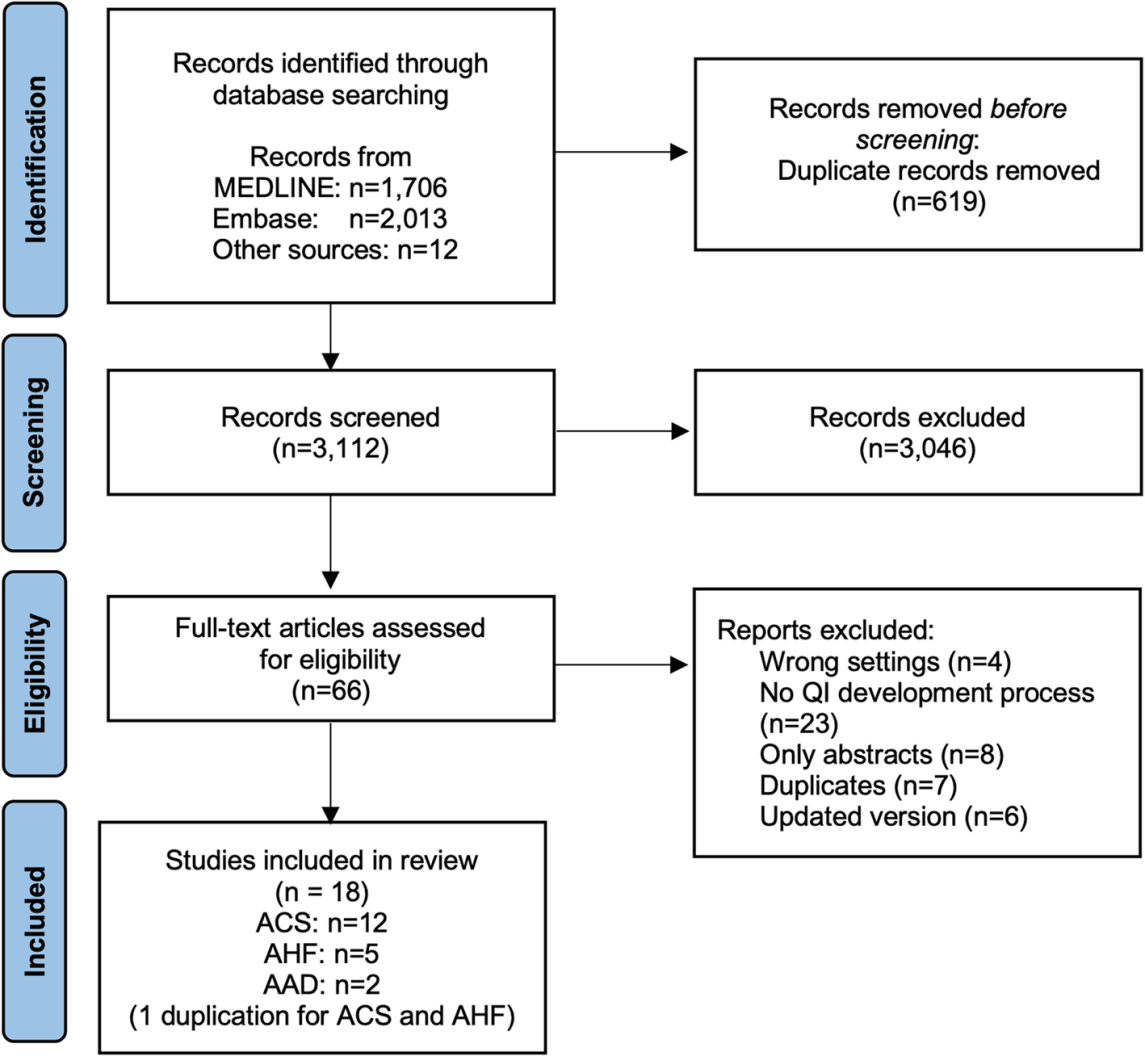
PRISMA flow diagram. PRISMA, Preferred Reporting Items for Systematic Reviews and Meta-Analyses; QI, quality indicator; ACS, acute coronary syndrome; AAD, acute aortic dissection; AHF, acute heart failure

Two reviewers (KK and YI) independently conducted the literature search. Any disagreements on study selection were resolved through discussion. The reasons for the exclusion of studies in the full-review process are presented in a flow diagram (Fig. 1).

### Data extraction and synthesis

Two researchers conducted data extraction using a standardized data collection form. First, a summary of each study, including the name of the first author, publication year, study setting (countries of origin), target disease, review and consensus-making process, and the number of QIs, was created. Second, the details of the QIs, including clinical setting, definition of QI, and number of publications cited, were summarized according to the predefined protocol [13]. Any disagreements were resolved by discussion, and if this failed, a resolution was reached through a third researcher (YM). For diseases with a large number of QIs, the most common QIs are listed in the Results section, and all items are presented in the Supporting Information materials. After a discussion, it was decided that similar QIs would be considered as one QI.

## Results

### Summary of publications

**Fig. 2:**
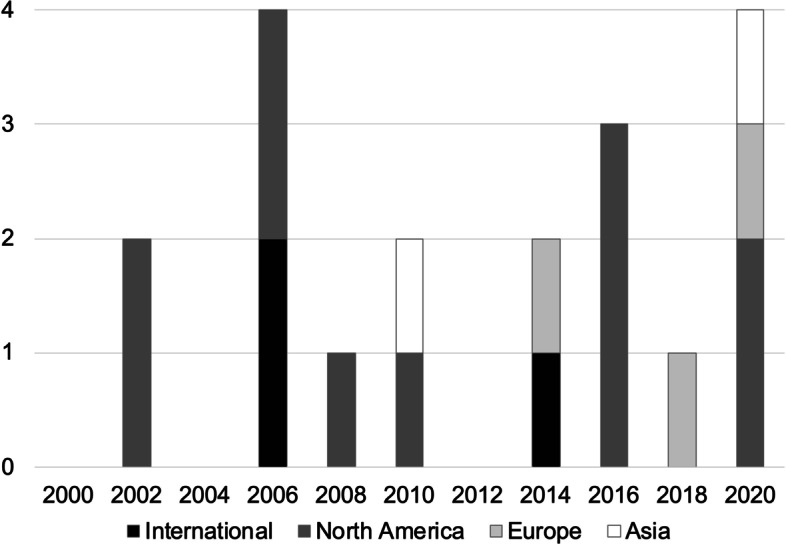
Summary of publication of quality indicators for acute cardiovascular diseases

Among the 3112 articles screened, 18 articles reporting search and creation processes were included in this scoping review (Fig. 1). Most of the guidelines for each acute cardiovascular disease were not included, as they did not mention how to create QI sets. Table 1 summarizes the characteristics of the included studies. Among the 18 included articles, 12 were on ACS, five on AHF, and two on AAD; one study included QIs for both ACS and AHF [11, 12, 19–34]. Of all articles, 11 articles were published in North America, and the number of publications differed among different years of publication (Fig. 2). Twelve articles were published after 2010 [11, 12, 19–25, 30, 33, 34]. Four studies conducted a systematic review [19, 26, 33, 34], and most of the studies used literature reviews that included a search for existing guidelines or statements. Ten studies used the Delphi or modified Delphi method in the QI creation process [11, 19, 22, 24–26, 28, 29, 32, 34]. Five studies conducted by cardiovascular societies or a government agency used a consensus-making process, based on their original protocols [12, 21, 23, 31]. Furthermore, four studies used expert panel consensus through discussion [21, 27, 31, 33]. We identified 268 QIs in the 18 evaluates articles, including the duplications between them: 191 QIs for ACS, 57 QIs for AHF, and 20 QIs for AAD.

**Table 1:** Summary of publications

No	Author	Year	Country/region	Review process	Consensus-making process	No. of QIs
Acute coronary syndrome						
1	Schiele et al. [11]	2021	Europe	Review	Modified Delphi method	26
2	Aeyels et al. [19]	2018	Belgium	Systematic review^a^	Delphi method	25
3	Jneid et al. [20]	2017	United States	Review	Defined by AHA guideline	17
4	Quraishi et al. [21]	2016	Canada	Review	Expert panel consensus	4
5	McNamara et al. [22]	2015	International	Review	Modified Delphi method	15
6	NICE (government agency) [23]	2014	United Kingdom	Review	Defined by NICE guideline	6
7	Sun et al. [24]	2011	China	Review	Modified Delphi method	23
8	Peña et al. [25]	2010	United States	Review	Modified Delphi method	10
9	Tu et al. [26]	2008	Canada	Systematic review^a^	Modified Delphi method	25
10	Watson et al. [27]	2007	United States	Review	Expert panel consensus	13
11	Idänpään-Heikkilä et al. [28]	2006	International	Review	Modified Delphi method	4
12	Tran et al. [29]	2003	Canada	Review	Modified Delphi method	23
Acute heart failure						
1	Heidenreich et al. [12]	2020	United States	Review	Defined by AHA guideline	8
2	McKelvie et al. [30]	2016	Canada	Review	Defined by CCS guideline	6
3	Heidenreich et al. [31]	2007	United States	Review	Expert panel consensus	11
4	Idänpään-Heikkilä et al. [28]	2006	International	Review	Modified Delphi method	3
5	Lee et al. [32]	2003	Canada	Review	Modified Delphi method	29
Acute aortic dissection						
1	Hassan et al. [33]	2021	Canada	Systematic review^a^	Expert panel consensus	11
2	Yamaguchi et al. [34]	2020	Japan	Systematic review^a^	Delphi method	9

*QI* quality indicator, *NICE* National Institute for Health and Care Excellence, *AHA* American Heart Association, *CCS* Canadian Cardiovascular Society

^a^A systematic review was defined as a review that included a search strategy

### QIs for ACS

QIs for ACS are listed in Table 2 (QIs mentioned in more than half [≥6] of the articles) and S1 Table (all-QI list). Most of the QIs (85%, *n* = 45) were process measures, and seven (13%) QIs were structural measures. The most mentioned measures were “time for primary percutaneous coronary intervention (PCI)/timely performed PCI” (*n* = 9), “beta-blockers prescription for patients with reduced left ventricular (LV) function” (*n* = 8), and “angiotensin-converting enzyme inhibitor (ACEi) or angiotensin II receptor blocker (ARB) prescription for patients with reduced LV function” (*n* = 8). Most of the process QIs were mentioned in ≤3 articles, and the process measures varied across studies and clinical settings (upon admission, acute setting, and during hospitalization/at discharge) (Table 3). Recent articles have referred to process measures for patient psychological and social factors, such as patient-reported health status and patient feedback [35]. For outcome measures, mortality or readmission were mentioned in seven articles. Among the 54 QIs, 26 (48%) were mentioned in only one article each. Among the structural indicators, the prehospital electrocardiogram was the most recommended indicator (*n* = 3).

**Table 2:** Commonly adopted quality indicators for ACS

Quality indicator	Clinical setting	Donabedian framework	Definition of quality indicator (representative)	No. of publications [reference]
Aspirin on arrival	Upon admission	Process	Patients were prescribed aspirin at arrival/patients with ACS	7 [19, 20, 24, 26–29]
Time for primary PCI/timely performed PCI	Acute setting	Process	Time from first medical contact or admission to primary PCI/timely PCI for STEMI or NSTEMI	9 [11, 20, 21, 23, 24, 26–29]
Time for fibrinolytic therapy	Acute setting	Process	Patients underwent < 10 min in case of reperfusion with fibrinolysis	6 [11, 20, 24, 26, 28, 29]
Aspirin at discharge	During hospitalization / at discharge	Process	Patients were prescribed aspirin at discharge/patients with ACS	6 [19, 20, 24–26, 29]
High-intensity statins prescription	During hospitalization / at discharge	Process	Patients were prescribed high-intensity statins/patients with ACS	7 [11, 19, 20, 24, 26, 27, 29]
Beta-blocker prescription	During hospitalization / at discharge	Process	Patients were prescribed beta-blockers/patients with reduced LV function	8 [11, 19, 20, 24–27, 29]
ACEi/ARB prescription	During hospitalization / at discharge	Process	Patients were prescribed ACEi or ARBs/patients with reduced LV function	8 [11, 19, 20, 24–27, 29]
LVEF assessment	During hospitalization / at discharge	Process	Patients who underwent assessment of LV function/patients with ACS	6 [11, 19, 20, 24, 26, 27]
Mortality or readmission	–	Outcome	Short- (30-day) or long-term mortality for hospitalized patients with ACS	7 [11, 21, 22, 24, 26, 28, 29]

*PCI* percutaneous coronary intervention, *ACS* acute coronary syndrome, *STEMI* ST elevation myocardial infarction, *NSTEMI* non-ST elevation myocardial infarction, *LV* left ventricular, *ACEi* angiotensin-converting enzyme inhibitor, *ARB* angiotensin II receptor blocker, *LVEF* left ventricular ejection fraction

**Table 3:** Variation of process measures according to the clinical settings in acute coronary syndrome and acute heart failure

Clinical settings	Number of publications	Number of QIs	Examples of QI
Acute coronary syndrome			
Upon admission	≥6	1	Aspirin at arrival
	4–5	1	Assessment of cardiovascular risk factors
	2–3	4	Assessment of 12 lead ECG, P2Y12 inhibitors before PCI.
	1	7	Registration of start of symptoms, assessment of cardiovascular antecedents.
Acute setting	≥6	2	Time for primary PCI/Timely performed PCI, time for fibrinolytic therapy.
	4–5	0	
	2–3	2	Early beta-blockers use, immediate angiography for cardiac arrest.
	1	9	Peri-procedural admission of morphine or alike, radial access.
During hospitalization / at discharge	≥6	5	Aspirin at discharge, high-intensity statins prescription.
	4–5	3	P2Y12 inhibitors at discharge, cardiac rehabilitation.
	2–3	4	Hypertension control, risk stratification with noninvasive stress testing.
	1	7	Mention about DAPT duration, provision of nutritional advice.
Acute heart failure			
Acute setting	≥3	0	
	2	1	Chest radiograph or another diagnostic test
	1	2	Medical history documentation, physical examination
During hospitalization / at discharge	≥3	4	Beta-blocker therapy for HFrEF, ACE inhibitor, ARB or ARNI therapy for HFrEF.
	2	2	Daily assessment of blood chemistry levels, post-discharge appointment.
	1	11	ARNI therapy for HFrEF, MRA therapy for HFrEF.

*QI* quality indicator, *ECG* electrocardiogram, *PCI* percutaneous coronary intervention, *DAPT* dual antiplatelet therapy, *HFrEF* heart failure with reduced ejection fraction, *ACE* angiotensin-converting enzyme inhibitor, *ARB* angiotensin II receptor blocker, *ARNI* angiotensin receptor-neprilysin inhibitor, *MRA* mineralocorticoid receptor antagonist

### Quality indications for AHF

QIs for AHF are listed in Table 4 (QIs mentioned in more than half [≥3] of the articles) and S2 Table (all-QI list). Most QIs (83%, *n* = 20) were process measures, and two (8%) were structural measures. The most mentioned measures were “ACEi, ARB, or angiotensin receptor neprilysin inhibitor therapy for patients with heart failure with reduced ejection fraction (HFrEF)” (*n* = 5) and “beta-blockers prescribed for patients with HFrEF” (*n* = 4). Outcome measures, such as mortality and readmission, were mentioned in three articles. Among the 24 QIs, 15 (60%) were mentioned in one article each. Among all the process measures (*n* = 20), 17 (85%) were used during the hospitalization / at discharge, and more than half of QIs were mentioned in only one article (Table 3).

**Table 4:** Commonly adopted quality indicators for acute heart failure

Quality indicator	Clinical setting	Donabedian framework	Definition of quality indicator (representative)	Number of publications [reference]
Beta-blocker therapy for HFrEF	During hospitalization / at discharge	Process	Patients prescribed beta-blocker therapy/patients with HFrEF	4 [12, 28, 31, 32]
ACE inhibitor, ARB or ARNI therapy for HFrEF	During hospitalization / at discharge	Process	Patients prescribed ACEi, ARB, or ARNI therapy/patients with HFrEF	5 [12, 28, 30–32]
Assessment of LV function	During hospitalization / at discharge	Process	Patients who underwent assessment of LV function/patients with HF	3 [30–32]
Patient education	During hospitalization / at discharge	Process	Percentage of patients with HF and family members who received education regarding HF management	3 [30–32]
Short or long-term mortality or readmission	–	Outcome	The proportion of mortality or HF readmission within 30 days or 1 year after discharge	3 [28, 30, 32]

*HFrEF* heart failure with reduced ejection fraction, *ACEi* angiotensin-converting enzyme inhibitor, *ARB* angiotensin II receptor blocker, *ARNI* angiotensin receptor neprilysin inhibitor, *LV* left ventricular, *HF* heart failure

### QIs for AAD

QIs for AAD are listed in Table 5. Two articles mentioned QIs for patients with AAD, and there was little overlap between the QIs listed in these articles [33, 34]. More than half of the QIs (58%, *n* = 7) were process measures, and four (33%) were structural measures. “Annual operation volume for AAD” was the sole indicator reported in both articles. One article included long-term measures such as follow-up imaging, mortality, and re-intervention. The other article included structural measures such as the designation of the emergency center and the number of surgeons or cardiologists.

**Table 5:** Quality indicators for AAD

Quality indicator	Clinical setting	Donabedian framework	Definition of quality indicator (representative)	Number of publications [reference]
Aortic dissection team	–	Structure	Presence of a dedicated institutional aortic dissection team	1 [33]
Emergency center	–	Structure	Designation of emergency center	1 [34]
Annual volume (open surgery or TEVAR)	–	Structure	Number of operations (open surgery or TEVER) per hospital or per surgeon	2 [33, 34]
No. of cardiovascular surgeons/cardiologists	–	Structure	Number of cardiovascular surgeons/board-certified cardiologists	1 [34]
Emergency computed tomography	Acute setting	Process	Patients who underwent emergency CT/AAD patients	1 [34]
Time to diagnosis/operation room	Acute setting	Process	Time from presentation to diagnosis/time from diagnosis to operation room	1 [33]
Use of hypothermic circulatory arrest	Acute setting	Process	Use of cardiopulmonary bypass technique involving cooling, stopping blood circulation, and antegrade brain perfusion	1 [33]
Intraoperative TEE	Acute setting	Process	Patients who underwent intraoperative TEE/AAD patients who underwent operative treatment	1 [34]
Blood pressure control by arterial line	Acute setting	Process	Patients who underwent arterial line/AAD patients	1 [34]
Beta-blocker use	Acute setting	Process	Beta-blocker use/AAD patients	1 [34]
1- year follow-up imaging	Chronic setting	Process	Number of performed CT/MRI studies with contrast /AAD patients	1 [33]
Short and long-term mortality/stroke/re-intervention	–	Outcome	Risk-adjusted 30-day or 1-year mortality/30-day stroke/1-year re-intervention following repair of type A AAD	1 [33]

*TEVAR* thoracic endovascular aortic repair, *CT* computed tomography, *AAD* acute aortic dissection, *TEE* transesophageal echocardiography, *MRI* magnetic resonance imaging

## Discussion

In this scoping review, we systematically reviewed the literature, evaluated the QI-developing process, and revealed the details of published QIs for acute cardiovascular diseases based on currently available evidence. We have revealed the following: (1) few of the articles conducted a systematic search with a search strategy; (2) there were many QI sets for ACS, but only five for AHF and two for AAD; and (3) QI measurements varied across articles, and each study defined its own QI measurements.

### QI development process

This is the first scoping review to systematically review the reporting quality of literature on QIs for acute cardiovascular diseases. This review revealed that few articles conducted a systematic search and provided a search strategy. Most of the articles on ACS and AHF performed a literature review without a search strategy, and performed a review of guidelines and associated literature. Except for one article [26], all of the literature that included systematic reviews was published after 2018. This implies that systematic reviews have been increasingly used to screen candidates for QIs in recent years.

We revealed that most of the studies that used a predefined creation process employed well-established methods for QI creation. In original research conducted by individual researchers, the Delphi or modified Delphi methods were frequently used. Although it is not clear which method is best to use, these systematic methods for decision making were recommended and widely used for QI development in healthcare [36]. In contrast, cardiovascular societies, such as the American Heart Association, and government agencies and the National Institute for Health and Care Excellence, published and used methodology papers to develop QIs. Moreover, these methods were also used for other cardiovascular diseases.

### Number of articles according to each cardiovascular disease

We found that the number of reports varied according to cardiovascular disease. There were more articles on ACS than on AHF and AAD. QIs for ACS have been reported for a longer period, and the list of QIs reported in the guidelines (American Heart Association/American College of Cardiology or European Society of Cardiology) has been updated depending on the care situation [35, 37–40]. Compared to ACS and AHF, all articles on AAD were published after 2020 [33, 34]. The number of QI sets may differ because the prevalence of ACS and AHF was higher, and QI sets for ACS and AHF have been reported since the 2000s; therefore, there was a large number of relevant studies. Additionally, the literature listed in our study was used for the assessment of the variation of care and showed the association with better outcome in other studies [41–43]. However, AAD is critical but infrequent, which explains the small number of relevant studies; thus, the validation of QI sets in AAD was limited.

### Variation of QI measurements for each cardiovascular disease

We found that QI measurements for each acute cardiovascular disease varied across articles. Most of the various QI measurements were categorized as process measurements. This finding is consistent with those of previous reviews on non-cardiovascular diseases [44, 45]. The QI measurements in our study were used in a variety of situations (upon admission, acute setting, during hospitalization / at discharge). Moreover, they included various factors, such as patient assessment, medical and surgical treatment, clinical tests, and patient education. In a recent update, novel QI measurements, such as the dual antiplatelet therapy duration and patient Quality of Life, were established as new QIs for ACS. This shows that emphasis is placed on both the acute and chronic care for patients with ACS. In addition, although we summarized outcome measures as a single category according to disease, outcome measures included a wide variety of definitions of the follow-up period and readmission outcomes, including ACS, AHF, and other causes.

Among all QI measurements, the most mentioned were “time for primary PCI/timely performed PCI” for ACS and “ACEi, ARB, or angiotensin receptor neprilysin inhibitor therapy for patients HFrEF” for AHF. These measurements have been reported since 2000, and a consensus was made on their use because most evaluated articles reported the same measurements. Commonly adopted QIs, shown in Table 2 for ACS and in Table 4 for AHF, were mentioned in more than half of the evaluated articles; these QIs are widely recommended in clinical settings.

We also found that there was wide variation in relation to the study setting (year of publication and country), QI construction process, and selection of QIs, among the different articles included in our study. Our scoping review did not focus on exploring the reasons underlying such variations; however, differences in literature searches, treatment strategies, patient backgrounds, and regional characteristics in each clinical setting may play a role in the variation of QIs. In this study, we provided a comprehensive list of QIs for acute cardiovascular diseases and clarified the commonly mentioned QIs for ACS and AHF. These consensus QI lists based on the assessment of the QI development process could be informative in the development of future QIs. Additionally, the use of commonly mentioned QIs may lead to improved outcomes related to the management of cardiovascular diseases.

### Study limitations

This study had several limitations. First, we did not evaluate the quality of the QI development methodologies because there are no established tools for such evaluation. However, we assessed a part of the QI development process and whether systematic search with search formulas and established QI creation processes were used. Second, QI sets were created according to the clinical setting, and we did not evaluate the creation process in detail. Finally, this was a scoping review with a synthesis approach, and we did not perform a detailed analysis of each original publication.

## Conclusion

This scoping review explicated the QI-making process and details of the currently published QIs for acute cardiovascular diseases. The study revealed that few of the articles conducted a systematic search using a search strategy, QI measurements varied across articles, and most QI sets were for ACS. We studied the most reported QIs, and the findings from this study will be useful to clinicians and organizations seeking to develop QI sets for acute cardiovascular care in the future.

## Supplementary Information

**Additional file 1: S1 Appendix.** MEDLINE (PubMed) search strategy. **S2 Appendix.** EMBASE (Dialog) search strategy. **S1 Table.** All quality indicators for acute coronary syndrome. **S2 Table.** All quality indicators for acute heart failure.

## Data Availability

All data generated during this research are incorporated in the article and its online supplementary material.

## Abbreviations

ACS: acute coronary syndrome
AHF: acute heart failure
AAD: acute aortic dissection
QI: quality indicator
JBI: Joanna Briggs Institute
PCI: percutaneous coronary intervention
LV: left ventricular
ACEi: angiotensin-converting enzyme inhibitor
ARB: angiotensin II receptor blocker
HFrEF: heart failure with reduced ejection fraction
