# Supplementary material for: Quality indicators for acute cardiovascular diseases: a scoping review
Source: BMC Health Serv Res. 2022 Jul 5;22:862. doi: 10.1186/s12913-022-08239-0 (PMC9254543; doi:10.1186/s12913-022-08239-0)
Supplement: Supplementary file 1 — Additional file 1: S1 Appendix. MEDLINE (PubMed) search strategy. S2 Appendix. EMBASE (Dialog) search strategy. S1 Table. All quality indicators for acute coronary syndrome. S2 Table. All quality indicators for acute heart failure. [file 12913_2022_8239_MOESM1_ESM.docx]

**Supporting information**

**Supplementary methods**

**S1 Appendix. MEDLINE (PubMed) search strategy**

# 1 "Myocardial Infarction/therapy"[Mesh Terms]

# 2 "Acute Coronary Syndrome/therapy"[ Mesh Terms]

# 3 "Heart Failure/therapy"[Mesh Terms]

# 4 "Aortic Diseases/therapy"[Mesh Terms]

# 5 "Aneurysm, Dissecting/therapy"[Mesh Terms]

# 6 #1 OR #2 OR #3 OR #4 OR #5

# 7 myocardial infarction*[tiab]

# 8 acute coronary syndrome*[tiab]

# 9 heart failure*[tiab]

# 10 aortic disease*[tiab]

# 11 aortic dissection*[tiab]

# 12 aortic aneurysm*[tiab]

# 13 #7 OR #8 OR #9 OR #10 OR #11 OR #12

# 14 #6 OR #13

# 15 "Quality Assurance, Health Care"[MeSH:NoExp]

# 16 "Quality Indicators, Health Care"[MeSH:NoExp]

# 17 "Outcome and Process Assessment, Health Care"[Mesh:NoExp]

# 18 #15 OR #16 OR #17

# 19 "organization and administration"[sh:NoExp]

# 20 standards[sh]

# 21 methods[sh]

# 22 #19 OR #20 OR #21

# 23 #18 AND #22

# 24 quality indicator*[tiab]

# 25 quality assessment tool*[tiab]

# 26 performance measure*[tiab]

# 27 #24 OR #25 OR #26

# 28 #23 OR #27

# 29 #14 AND #28

**S2 Appendix. EMBASE (Dialog) search strategy**

# 1 EMB.EXACT.EXPLODE("heart infarction -- therapy")

# 2 EMB.EXACT.EXPLODE("heart infarction -- drug therapy")

# 3 EMB.EXACT.EXPLODE("heart infarction -- surgery")

# 4 EMB.EXACT.EXPLODE("heart infarction -- radiotherapy")

# 5 EMB.EXACT.EXPLODE("heart infarction -- disease management"))

# 6 EMB.EXACT.EXPLODE("acute coronary syndrome -- drug therapy")

# 7 EMB.EXACT.EXPLODE("acute coronary syndrome -- radiotherapy")

# 8 EMB.EXACT.EXPLODE("acute coronary syndrome -- surgery")

# 9 EMB.EXACT.EXPLODE("acute coronary syndrome -- therapy")

# 10 EMB.EXACT.EXPLODE("acute coronary syndrome -- disease management"))

# 11 EMB.EXACT.EXPLODE("heart failure -- radiotherapy"

# 12 EMB.EXACT.EXPLODE("heart failure -- therapy")

# 13 EMB.EXACT.EXPLODE("heart failure -- drug therapy")

# 14 EMB.EXACT.EXPLODE("heart failure -- surgery")

# 15 EMB.EXACT.EXPLODE("heart failure -- disease management"))

# 16 EMB.EXACT.EXPLODE("aortic disease -- surgery")

# 17 EMB.EXACT.EXPLODE("aortic disease -- radiotherapy")

# 18 EMB.EXACT.EXPLODE("aortic disease -- therapy")

# 19 EMB.EXACT.EXPLODE("aortic disease -- drug therapy")

# 20 EMB.EXACT.EXPLODE("aortic disease -- disease management")

# 21 EMB.EXACT.EXPLODE("dissecting aneurysm -- drug therapy")

# 22 EMB.EXACT.EXPLODE("dissecting aneurysm -- surgery")

# 23 EMB.EXACT.EXPLODE("dissecting aneurysm -- therapy")

# 24 EMB.EXACT.EXPLODE("dissecting aneurysm -- radiotherapy")

# 25 EMB.EXACT.EXPLODE("dissecting aneurysm -- disease management")

# 26 #1 OR #2 OR #3 OR #4 OR #5 OR #6 OR #7 OR #8 OR #9 OR #10 OR #11 OR #12 OR #13 OR #14 OR #15 OR #16 OR #17 OR #18 OR #19 OR #20 OR #21 OR #22 OR #23 OR #24 OR #25

# 27 TI,AB(myocardial P/0 infarction*)

# 28 TI,AB (acute P/0 coronary P/0 syndrome*)

# 29 TI,AB (heart P/0 failure*)

# 30 TI,AB (aortic P/0 (disease* OR dissection* OR aneurysm*))

# 31 #27 OR #28 OR #29 OR #30

# 32 #26 OR #31

# 33 MJEMB.EXACT("performance measurement system")

# 34 MJEMB.EXACT("health care quality")

# 35 #33 OR #34

# 36 TI,AB(quality P/0 indicator*)

# 37 TI,AB (quality P/0 assessment P/0 tool*)

# 38 TI,AB (performance P/0 measure*)

# 39 #36 OR #37 OR # 38

# 40 #35 OR #39

# 41 #32 AND #40

**S1 Table. Quality indicators for acute coronary syndrome**

| Quality indicator | Clinical setting | Donabedian framework | Definition of quality indicator (representative) | Number of publications |
| --- | --- | --- | --- | --- |
| PCI Network/primary PCI center | – | Structure | Participation in network organization for the management of ACS | 2 |
| Participation in quality program or registry | – | Structure | Participation in a registry for quality assessment | 2 |
| Availability of hs-cTn | – | Structure | High-sensitivity cardiac troponin | 1 |
| Prehospital ECG | – | Structure | Available for prehospital interpretation of ECG | 3 |
| Systematic assessment of time to reperfusion | – | Structure | Systematic assessment of time to reperfusion | 1 |
| Hospitals available for emergency thrombolysis | – | Structure | Hospitals available for emergent thrombolysis therapy after a call for emergency medical services | 2 |
| Annual PCI volume | – | Structure | Annual PCI volume per center or provider | 1 |
| Registration of start of symptoms | Upon admission | Process | Registration of onset of symptoms | 1 |
| Assessment of cardiovascular risk factors | Upon admission | Process | Assessment of cardiovascular risk factors (hypertension, diabetes, including smoking, etc.) | 4 |
| Assessment of cardiovascular antecedents | Upon admission | Process | Assessment of cardiovascular antecedents | 1 |
| Assessment of prehospital medication and allergies | Upon admission | Process | Assessment of prehospital medication and allergies | 1 |
| Diagnosis of acute myocardial infarction | Upon admission | Process | Proportion of adults with a diagnosis of acute myocardial infarction using the criteria in the universal definition of myocardial infarction. | 1 |
| Assessment of 12 lead ECG | Upon admission | Process | Assessment and interpretation of a 12-lead ECG | 3 |
| Assessment of sBP and weight | Upon admission | Process | Assessment of sBP and weight | 1 |
| Assessment of STEMI TIMI score | Upon admission | Process | Assessment of STEMI thrombolysis in myocardial infarction score | 1 |
| Assessment of blood test | Upon admission | Process | Assessment of blood tests (cardiac troponin, creatinine kinase etc.) | 2 |
| Assessment of reperfusion type | Upon admission | Process | Assessment of reperfusion type | 1 |
| Aspirin at arrival | Upon admission | Process | Patients were prescribed aspirin at arrival/patients with ACS | 7 |
| P2Y12 inhibitors before PCI | Upon admission | Process | Patients were prescribed P2Y12 inhibitors/patients with ACS required P2Y12 inhibitors | 3 |
| Peri-procedural admission of anticoagulant | Upon admission | Process | Peri-procedural anticoagulation (unfractionated heparin, etc.) upon (or before) admission | 2 |
| Peri-procedural admission of morphine or alike | Acute setting | Process | Peri-procedural admission of morphine or morphine-like drug | 1 |
| Early beta-blockers use | Acute setting | Process | Patients were prescribed beta-blockers within 12 hours of arrival B13/ patients with ACS without contraindication for beta-blockers | 3 |
| Time for primary PCI/Timely performed PCI | Acute setting | Process | Time from first medical contact or admission to primary PCI/timely PCI for STEMI or NSTEMI | 9 |
| Time for fibrinolytic therapy | Acute setting | Process | Patients underwent <10 min in case of reperfusion with fibrinolysis | 6 |
| Immediate angiography for cardiac arrest | Acute setting | Process | Immediate angiography for resuscitated out-of-hospital cardiac arrest in STEMI patients | 2 |
| Radial access | Acute setting | Process | Radial-first strategy for patients with ACS underwent PCI | 1 |
| ECG monitoring | Acute setting | Process | ECG monitoring (24–48 h) | 1 |
| PCI failure | Acute setting | Process | Emergent CABG for failed PCI | 1 |
| Significant dissection/perforation | Acute setting | Process | Angiographic or clinical evidence of perforation | 1 |
| Acute renal failure | Acute setting | Process | New requirement for dialysis | 1 |
| Stroke | Acute setting | Process | Patients with ischemic or hemorrhagic stroke after PCI | 1 |
| Vascular complications | Acute setting | Process | Vascular complications at percutaneous entry site | 1 |
| Bleeding event | Acute setting | Process | Bleeding events within 72 hours after PCI | 1 |
| Aspirin at discharge | During hospitalization / at discharge | Process | Patients were prescribed aspirin at discharge/patients with ACS | 6 |
| P2Y12 inhibitors at discharge | During hospitalization / at discharge | Process | Patients were prescribed a P2Y12 inhibitor at discharge/patients with ACS | 4 |
| Mention about DAPT duration | During hospitalization / at discharge | Process | Shortening or prolongation of the DAPT duration at discharge | 1 |
| High-intensity statins prescription | During hospitalization / at discharge | Process | Patients were prescribed high-intensity statins/patients with ACS | 7 |
| Beta-blocker prescription | During hospitalization / at discharge | Process | Patients were prescribed beta-blockers/patients with reduced LV function | 8 |
| ACEi/ARB prescription | During hospitalization / at discharge | Process | Patients were prescribed ACEi or ARBs/patients with reduced LV function | 8 |
| ACEi/ARB prescribed in diabetes with renal failure | During hospitalization / at discharge | Process | Patients were prescribed ACEi or ARBs/patients with diabetes with renal failure | 1 |
| LVEF assessment | During hospitalization / at discharge | Process | Patients received an assessment of LV function/patients with ACS | 6 |
| Hypertension control | During hospitalization / at discharge | Process | Patients achieved lower blood pressure control before discharge | 2 |
| Cardiac rehabilitation (during admission or referral) | During hospitalization / at discharge | Process | Patients with cardiac rehabilitation referral or underwent rehabilitation/patients with ACS | 5 |
| Risk stratification with noninvasive stress testing | During hospitalization / at discharge | Process | Noninvasive stress testing, perfusion imaging, or stress echocardiography before discharge in conservatively treated patients | 3 |
| Patient feedback | During hospitalization / at discharge | Process | Feedback regarding the patient’s experience | 1 |
| Smoking cessation counseling | During hospitalization / at discharge | Process | Smoking cessation counseling/patients with ACS | 5 |
| Provision of nutritional advice | During hospitalization / at discharge | Process | Complete lifestyle changes (nutritional advice and physical activity) | 1 |
| Quality of life/patient-reported health status | During hospitalization / at discharge | Process | Patients with systematic assessment of health-related quality of life/patients with ACS | 2 |
| Discharge letter | During hospitalization / at discharge | Process | Copying the hospital discharge letter to the patient | 1 |
| Evaluation for depression | During hospitalization / at discharge | Process | Depression screening within 3 months for patients with ACS | 1 |
| Length of intensive care unit | During hospitalization / at discharge | Process | Length of intensive care unit stay | 1 |
| Length of hospital stay | During hospitalization / at discharge | Process | Length of hospital stay (all or post-procedural) | 2 |
| Mortality or readmission | – | Outcome | Short- (30-day) or long-term mortality for hospitalized patients with ACS | 7 |

PCI, percutaneous coronary intervention; ACS, acute coronary syndrome; hs-cTn, high sensitive- cardiac troponin; ECG; electrocardiogram; sBP, systolic blood pressure; STEMI, ST elevation myocardial infarction; NSTEMI, non-ST elevation myocardial infarction; CABG, coronary artery bypass grafting; DAPT, dual antiplatelet therapy; LV, left ventricular; ACEi, angiotensin-converting enzyme inhibitor; ARB, angiotensin II receptor blocker; LVEF, left ventricular ejection fraction; TIMI, thrombolysis in myocardial infarction

**S2 Table. All quality indicators for acute heart failure**

| Quality indicator | Clinical setting | Donabedian framework | Definition of quality indicator (representative) | Number of publications |
| --- | --- | --- | --- | --- |
| Registry/program participation | – | Structure | Participation in a national or regional heart failure registry that provides regular performance reports based on benchmarked data | 2 |
| Coding accuracy of heart failure discharge abstracts | – | Structure | The quality of CHF coding determined by the proportion of patients who qualify as CHF cases based on clinical criteria | 1 |
| Medical history documentation | Acute setting | Process | Patients with documented symptoms or medical history/patients newly diagnosed as HF | 1 |
| Physical examination | Acute setting | Process | Patients who underwent and documented the results of physical examination/patients newly diagnosed as HF | 1 |
| Chest radiograph or another diagnostic test | Acute setting | Process | Patients who underwent chest radiography/patients admitted to the hospital | 2 |
| Beta-blocker therapy for HFrEF | During hospitalization / at discharge | Process | Patients were prescribed beta-blocker therapy/patients with HFrEF | 4 |
| ACE inhibitor, ARB or ARNI therapy for HFrEF | During hospitalization / at discharge | Process | Patients were prescribed ACEi, ARB or ARNI therapy/patients with HFrEF | 5 |
| ARNI therapy for HFrEF | During hospitalization / at discharge | Process | Patients were prescribed ARNI therapy/patients with HFrEF and remained symptomatic after prescription of ACEi or ARB | 1 |
| MRA therapy for HFrEF | During hospitalization / at discharge | Process | Patients were prescribed MRA therapy/patients with HFrEF and symptomatic despite treatment with beta-blockers, ACEi, ARB, or ARNI | 1 |
| Hydralazine/isosorbide dinitrate therapy for HFrEF | During hospitalization / at discharge | Process | Patients were prescribed beta-blocker therapy/patients with HFrEF and are self-identified as Black or African American and receiving ACEi, ARB, or ARNI and beta-blocker | 1 |
| Anticoagulants for AF | During hospitalization / at discharge | Process | Patients were prescribed anticoagulants/patients with AF | 1 |
| Treatment without CCBs for HFrEF and no AF | During hospitalization / at discharge | Process | Patients were prescribed CCBs/patients with HFrEF and without AF | 1 |
| Treatment without antiarrhythmic agents for HFrEF | During hospitalization | Process | Patients were prescribed type 1 antiarrhythmic agents/patients with HFrEF | 1 |
| Digoxin monitoring | During hospitalization / at discharge | Process | Patients checked for digoxin level/patients prescribed digoxin | 1 |
| Weight monitoring | During hospitalization / at discharge | Process | Weights measured/recorded ≥50% of in-hospital days | 1 |
| Daily assessment of blood chemistry levels | During hospitalization / at discharge | Process | Patients who received electrolytes and underwent renal function assessment/patients hospitalized with HF | 2 |
| Laboratory monitoring in new MRA therapy | During hospitalization / at discharge | Process | Patients checked for potassium level and renal function within 1 week/patients started on MRA therapy | 1 |
| Assessment of LV function | During hospitalization / at discharge | Process | Patients who underwent an assessment of LV function/patients with HF | 3 |
| Patient education | During hospitalization / at discharge | Process | The percentage of patients with HF and family members who received education regarding HF management | 3 |
| Exercise training referral | During hospitalization / at discharge | Process | Patients referred for outpatient exercise training/patients with HFrEF | 1 |
| Post-discharge appointment | During hospitalization / at discharge | Process | Patients with a follow-up appointment scheduled within 7 days/patients with HF and discharged alive | 2 |
| Length of hospital stay | During hospitalization / at discharge | Process | Length of hospital stay | 1 |
| Short or long-term mortality or readmission | – | Outcome | The proportion of mortality or HF readmission within 30 days or one year after discharge | 3 |
| ED visits for CHF or any cardiovascular diseases | – | Outcome | ED visits for HF or other cardiovascular causes within 30 days or one year of discharge | 1 |

HFrEF, heart failure with reduced ejection fraction; ACEi, angiotensin-converting enzyme inhibitor; ARB, angiotensin II receptor blocker; ARNI, angiotensin receptor neprilysin inhibitor; MRA, mineralocorticoid receptor antagonists; AF, atrial fibrillation; CCB, calcium channel blocker; LV, left ventricular; ED, emergency department; CHF, congestive heart failure
